# Attitudes towards geroprotection: measuring willingness, from lifestyle changes to drug use

**DOI:** 10.3389/fragi.2024.1440661

**Published:** 2024-11-05

**Authors:** Sam J. Brouwers, Georges E. Janssens, Tali Spiegel

**Affiliations:** ^1^ Department of Sociology, Utrecht University, Utrecht, Netherlands; ^2^ Laboratory Genetic Metabolic Diseases, Amsterdam University Medical Centers, University of Amsterdam, Amsterdam, Netherlands; ^3^ Amsterdam Gastroenterology, Endocrinology and Metabolism Institute, Amsterdam University Medical Centers, Amsterdam, Netherlands

**Keywords:** exercise, supplements, intermittent fasting, metformin, rapamycin, prevention, healthy ageing, geroprotection

## Abstract

**Introduction:**

Geroprotection is an emerging field of research focused on devising strategies for combating the mechanisms of ageing. This study held three aims: 1) to explore the willingness to utilise five different geroprotective measures (i.e., exercise, supplements, intermittent fasting, metformin, and rapamycin use), 2) to explore whether the willingness differs based on respondents’ sociodemographic characteristics and 3) to explore the association between trust in medical institutions and willingness to utilise different geroprotective measures.

**Methods:**

A questionnaire was used to assess the attitudes of a sample of the Dutch population by way of both convenience and snowball sampling (final N = 178). Descriptive data and bivariate correlations were used in the analyses.

**Results:**

Relatively high social acceptance of both exercise (66%) and supplements (82%) was found, whereas intermittent fasting (30%), metformin (26%), and rapamycin (10%) were less supported. Males were significantly more likely to be open to exercise and women to supplement use. Trust in medical institutions correlated significantly with the willingness to start metformin.

**Discussion:**

Exploratory research can only provide a first step in understanding the social acceptance of geroprotection measures. Nevertheless, this study clearly illustrates more well-known measures promoted by public health policy are also more accepted and used. Public health campaigns could consider the sex differences in the uptake of exercise and supplements, and future research may want to delve deeper into the role of facilitating trust relations between medical institutions and the public in promoting the use of geroprotective drugs.

## 1 Introduction

Currently life expectancy at birth in the Netherlands is 80.1 for males, and 83.1 for females (Statistics Netherlands, 2023b). Alongside an increase in life expectancy, an increase in the prevalence of (multiple) chronic illnesses can be observed in individuals over 60 (Van Der Heide et al., 2018). Geroprotection is an emerging field of research combining physiology, nutrition, nutraceuticals, and medicine to devise strategies for combating the mechanisms of ageing (Magon, Chopra, and Kumar 2012). Utilising one or more geroprotective mechanisms may lead to a delay of the onset of chronic illness in older age (Morsli and Bellantuono, 2021). Alongside well-researched lifestyle factors such as exercise, research from animal models has revealed numerous small molecules as candidates to promote health, extend lifespan, and become the first true geroprotective drugs (Partridge et al., 2020).

The current study holds three aims. First, we explore which of five geroprotective measures are favoured by a sample of Dutch adults. The geroprotective measures included have all shown potential for disease prevention or improvement: exercise (Pedersen, 2019; Rebelo-Marques et al., 2018), supplements (Ames, 2018; Gupta and Prakash, 2015; van Rossum et al., 2020), intermittent fasting (de Cabo and Mattson, 2019; Longo et al., 2021), and the use of geroprotective drugs, namely; metformin (Partridge et al., 2020; Bannister et al., 2014; Claesen et al., 2016; Soukas, Hao, and Wu, 2019) and rapamycin (Selvarani et al., 2021; Zhang et al., 2021; Lamming, 2016). Of special interest is to explore whether individuals are as open to geroprotective drugs as to other lifestyle changes. Literature on the willingness of individuals to engage in geroprotective mechanisms suggests that when it comes to life extending drugs, just 35% would intend to take life-extension pills if they became available in the near future (Partridge et al., 2011). This is not surprising as geroprotective drugs do not always produce clear-cut positive findings (Keys et al., 2022), and hold potential side-effects for the consumer (Selvarani et al., 2021; Soukas et al., 2019; Lamming, 2016).

Second, this research assesses the relationship between respondents’ socio-demographic data and their willingness to implement geroprotective strategies into their daily lives. Literature suggests that geroprotective measures can lead to different medical outcomes for different segments in society. This is particularly prominent in the case of sex. For example, the same regimen of intermittent fasting can lead to different medical outcomes amongst men and women (Cienfuegos et al., 2022). Additionally, certain geroprotective measures are known to be more accessible to certain groups, such as observed lower physical activity levels amongst older and chronically ill individuals compared to healthy young individuals (Loef et al., 2016). Likewise, certain geroprotective compounds, such as metformin and rapamycin, show different lifespan effects when tested in model organisms (Bartke et al., 2019). More studies exploring the willingness of different segments of society in using geroprotective drugs is needed to produce impactful targeted public health policy.

Third, we aim to assess whether trust in various medical institutions impacts the willingness of individuals to the five different types of geroprotective mechanisms. Some studies suggest that people who are sceptical towards geroprotectors also report distrusting modern medicine and medical professionals (Smol’kin et al., 2018). The topic of trust in professionals and institutions in relation to the acceptance of new medical technology has been extensively studied in survey research about genetic engineering and innovative healthcare technology (Mohr et al., 2007; Priest et al., 2003; Calnan et al., 2005). Geroprotection, similar to the state of genetic engineering in the early 2000s, is poorly understood or known by the general public (Smol’kin et al., 2018). To cope with this lack of knowledge people may rely on social trust and trust in institutions to reduce the complexity of the risk management decisions involved in the acceptance of geroprotective strategies (Siegrist, 2000).

## 2 Materials and methods

### 2.1 Study population

A questionnaire was used to assess the attitudes of a sample of the Dutch population, 18 years and older, towards geroprotective measures. Specifically the following themes were addressed: demographic characteristics, health status, trust in medical institutions, and the social acceptance of geroprotective interventions. At the start of the questionnaire, subjects were informed about the research project’s purpose, the voluntary nature of participation, and the data and anonymity protocols that would be followed. All data were collected between 20 December 2022, and 31 March 2023, by way of both convenience and snowball sampling. The researchers involved shared the survey in their respective social networks by way of online platforms and requested their networks to share the survey further. Additionally, QR codes linked to the questionnaire were displayed in Utrecht University buildings and local libraries. The distribution resulted in an initial sample of 211 respondents, however, after excluding incomplete survey entries the final sample consisted of 178 respondents.

### 2.2 Measurements

*Geroprotective measures*. To make the results found in this research insightful and applicable in the short term, respondents were asked about specific geroprotective measures supported by research and experts. By using geroprotection methods which are currently studied rather than hypothetical future geroprotectors, respondents were able to evaluate most of the required time investment, effort, and possible side effects against the outlined health-related boons. This is an essential step in coupling geroprotection to real-world usability.

The intention to employ geroprotection was operationalized as the respondent’s willingness to adapt the described geroprotective measures into daily life. These consisted of exercise, supplement intake, intermittent fasting, metformin, and rapamycin use. This survey sought to carve a middle path between enthusiasts and sceptics of geroprotective technology. Thus, regarding exercise and supplementation, rather widely acknowledged examples of geroprotective benefits were shared in example texts. Meanwhile, on the subjects of intermittent fasting, metformin, and rapamycin, speculative statements were avoided (Supplementary material S1). The introductory texts used sought to strike a balance between providing sufficient information and remaining readable and comprehensible to avoid survey dropout. Thus, example texts consisted of two–eight sentences. After each short text participants were asked whether they already met the proposed level of engagement in the specific measure and whether they were willing to commit to the proposed engagement if they did not yet (ranging from 1: “certainly not” to 5: “certainly yes”). The specific proposed engagement for each geroprotective intervention was: *Exercise*: at least 3 h of aerobic exercise and 2 h of resistance training weekly. *Supplements*: a daily intake of an omega-3 supplement, or another supplement with verifiable health benefits. According to what the respondent perceived as verifiable health benefits. *Intermittent fasting*: defined as at least 5 days of 16/8 intermittent fasting weekly. *Metformin* and *rapamycin*: one pill weekly, provided the geroprotective agent yields health- and life-span-related benefits. According to what the respondent perceived as health- and life-span-related benefits.

This survey’s analysis is based on the willingness and intentions of respondents. A respondent who already met the proposed behaviour was considered to be similar to a respondent who answered “certainly yes” to a given intervention. Thus, both participants would be assigned the value “5” for that variable. For both metformin and rapamycin, no measure of current use was included, therefore this data manipulation was not present for those variables.

The *demographic characteristics* used in this study include: *Gender*: three options were included (0: “male”, 1: “female”, and 2: “other”). In the correlation analyses, gender was dichotomised (0: “male”, and 1: “female”). *Age*: measured as a continuous variable (in years). However, to conduct an exhaustive analysis of the effect of age, both a numerical and categorical version of the variable were added. *Chronic condition*: whether or not respondents were subject to a chronic health condition or an incurable disease was measured by three options (0: “no”, 1: “yes”, or 2: “I won’t say”). For this analysis, these were not further defined (e.g., physical/psychological, or minor/severe), and in the correlation analyses, the chronic condition variable was dichotomized (0: “no”, and 1: “yes”). *Subjective health*: ascertained by asking respondents to rate their health on a 5-point scale (1: “poor”, 2: “mediocre”, 3: “fair”, 4: “good”, or 5: “excellent”).

*Trust in medical institutions* was operationalized by combining four 5-point Likert scale questions (ranging from 1: “no trust” to 5: “very much trust”) about respondents’ trust in scientists and researchers at universities, pharmaceutical companies, doctors and hospital personnel, and the participants’ general practitioner and general practice. In designing the survey we realised trust is a multifaceted, complex subject. In previous research, both trust in healthcare practitioners and research institutions were assessed to be connected to the social acceptance of innovative healthcare technologies (Smol’kin et al., 2018; Calnan et al., 2005; Siegrist, 2000). Therefore we chose to combine these facets into a conceptually wider scale of trust in medical institutions. Participants were asked about “your general practitioner and general practice”, as this has been shown to affect trust differently compared to medical institutions in general (Calnan and Rowe, 2006). Only if all four items were answered the scale variable was constructed using the mean of all items combined.

### 2.3 Statistical analysis

**FIGURE 1:**
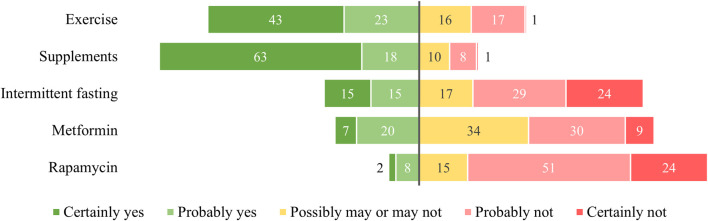
The willingness to start using geroprotective measures in percentages (%). Percentages were calculated using the Largest Remainder Method for rounding to 100%. *N* = 178.

**FIGURE 2:**
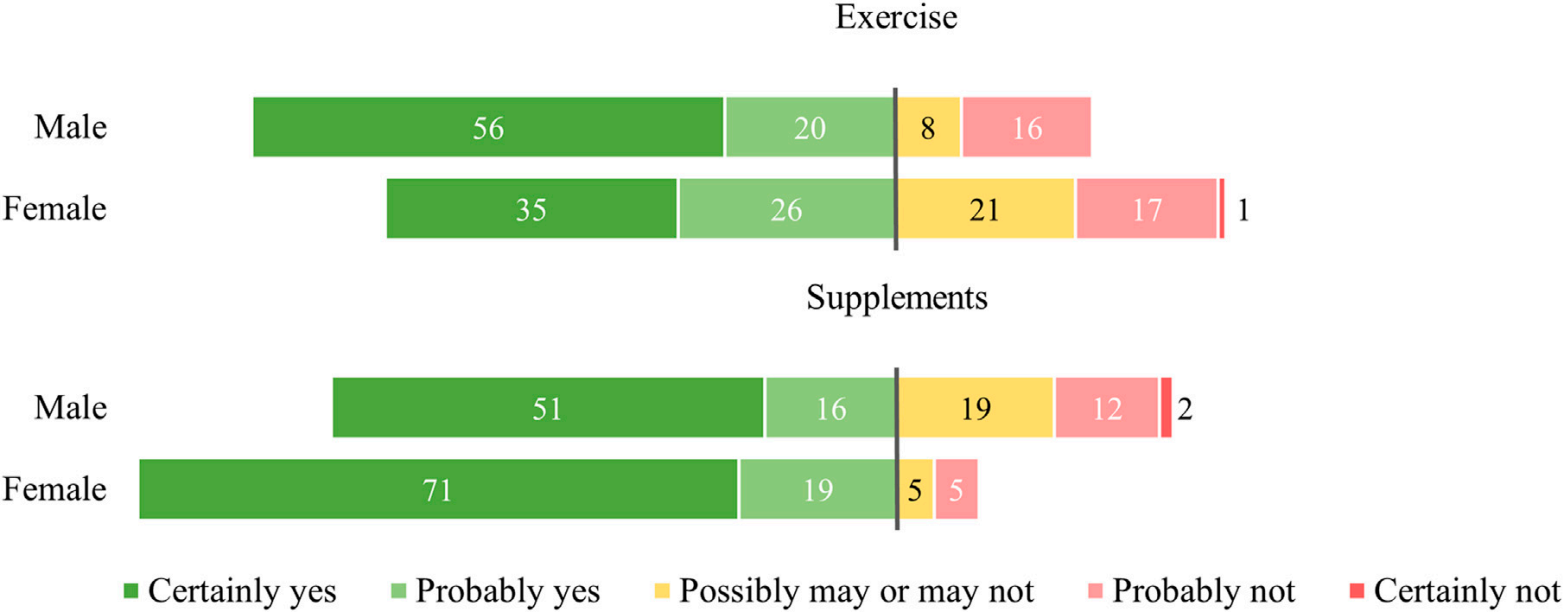
The willingness to start using the exercise regimen and supplements by gender in percentages (%). Percentages were calculated using the Largest Remainder Method for rounding to 100%. *N*= 178 (*N*_male_ = 64, *N*_female_ = 113).

For the exploratory research conducted in this paper, we utilised descriptive data on the willingness to use geroprotective measures (Figures 1, 2) and conducted bivariate analyses using the Pearson correlation coefficient (Table 2) in R Version 4.2.2. Therefore, in the correlation analyses subjective health was treated as a continuous variable (Schnittker and Bacak, 2014). In all analyses, a P-value of less than 0.05 was considered to indicate statistical significance.

## 3 Results

The socio-demographic characteristics of respondents are reported in Table 1. 63.5% (95% CI 56.1–70.3) were female, which overrepresents the mean 50.3% of females in the Netherlands (Statistics Netherlands, 2023a), and the sample disproportionally consists of younger groups (median in years 25, IQR 21–31; range 18–85). The discrepancy between the Dutch population average and the sample data should be considered in the evaluation of survey results. However, due to the exploratory nature of the study and sizable overrepresentation observed, we opted for acknowledgement of the confined generalizability of the data and the variables remained unweighted.

**TABLE 1:** Socio-demographic characteristics of the study population.

Characteristics	*n*	% (95% CI)
Gender		
Male	64	36.0 (29.2–43.3)
Female	113	63.5 (56.1–70.3)
Other	1	0.56 (0.08–3.95)
Age (categorical)		
18–24	88	49.4 (42.1–56.8)
25–39	57	32.0 (25.5–39.3)
40–54	22	12.4 (8.25–18.1)
55+	11	6.18 (3.44–10.9)
Subjective health		
1. Poor	0	0
2. Mediocre	3	1.69 (0.54–5.15)
3. Fair	43	24.2 (18.4–31.1)
4. Good	104	58.4 (51.0–65.5)
5. Excellent	28	15.7 (11.0–21.9)
Reported to have a chronic condition		
Yes	41	23.0 (17.4–29.9)
No	136	76.4 (69.5–82.1)
“I won’t say”	1	0.56 (0.08–3.95)

Notes: *N* = 178.

The average subjective health experience was assessed as slightly below “Good” (mean 3.88, SD 0.67). Additionally, the trust in medical institutions expressed by respondents ranged from below 3: “reasonable trust” in pharmaceutical companies (mean 2.79, SD 0.83), to above 4: “plenty trust” in scientists and researchers at universities (mean 4.13, SD 0.8). Overall, the trust in medical institutions scale shows sufficient consistency (Cronbach α = .76, 95% CI .70–.81; mean 3.68, SD 0.62), indicating the measure is reliable.

Figure 1 depicts the observed willingness to implement the five geroprotective strategies into daily life. Relatively high social acceptance, indicated by respondents stating they certainly or probably would adopt the measure, of both exercise (66.3%; 95% CI 59.0–72.9) and supplements (81.5%; 95% CI 75.0–86.6) is observed. The intermittent fasting schedule is less supported (29.8%; 95% CI 23.5–37.0), as is metformin (26.4%; 95% CI 20.4–33.4). Yet, a larger fraction of respondents expressed uncertainty (34.4% to 16.9%) and a relatively smaller group was willing to fully dismiss the measure (8.99% to 24.2%), indicating that respondents are “on the fence” when it comes to metformin use. Rapamycin use was the least accepted geroprotective strategy (9.55%; 95% CI 5.99–14.9).

The correlation analysis can be seen in Table 2. No association was found between the willingness to implement any of the geroprotective measures and subjective health, chronic illness, or age. The continuous and categorical versions of the age variable differed only in their significant (continuous age r = −.180, *p* = .017) or non-significant (categorical age r = −.124, *p* = .100) correlation with respondents’ trust in medical institutions. The association of sex with the willingness to implement the geroprotective measures was found to be multidimensional. As pictured in Figure 2, males were significantly more likely to be open to or already practising the exercise regimen (r = −.166, *p* = .027), while women were significantly more willing to use or were already using supplements (r = .245, *p* = .001).

**TABLE 2:** Correlation table.

Measure	Correlations								
	1	2	3	4	5	6	7	8	9
1. Gender (female)									
2. Age (numerical)	−0.013								
3. Subjective health	−0.150	−0.020							
4. Chronic condition	−0.003	0.003	−0.287						
5. Trust in MI	−0.076	−0.180	0.151	−0.092					
6. Exercise	−0.166	−0.044	0.030	0.116	−0.037				
7. Intermittent fasting	−0.065	0.050	−0.042	0.026	−0.081	0.178			
8. Supplements	0.245	0.003	−0.013	0.040	−0.036	0.058	0.205		
9. Metformin	−0.020	−0.126	0.102	−0.076	0.194	−0.030	0.234	0.185	
10. Rapamycin	−0.144	−0.115	0.122	0.042	0.033	0.163	0.128	0.092	0.574

Notes: 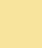 *p* < .05, 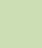 *p* < .01. *N* = 178. 1 respondent gender = “other” excluded in gender correlations and 1 respondent chronic condition = “I won’t say” excluded in chronic condition correlations.

Trust in medical institutions correlated significantly with the willingness to start metformin use (r = .194, *p* = .009), suggesting more trust in medical institutions is connected to increased acceptance of the candidate geroprotection drug. Lastly, multiple positive correlations exist between geroprotective strategies. The strongest of which is between the two repurposed medicines metformin and rapamycin (r = .574, *p* < .001).

## 4 Discussion

The current study held three aims: 1) to explore the willingness of a Dutch sample in utilising five different geroprotective measures, 2) to explore whether the willingness differs based on respondents’ sociodemographic characteristics and 3) to explore the association between trust in medical institutions and willingness to utilise different geroprotective measures. The findings suggest that the respondents’ willingness to engage in geroprotective strategies and measures varies widely between the different mediums. The more well-known and commonly practised measures of vigorous exercise, including both endurance and strength training, and daily use of supplements were accepted by more than two-thirds of respondents (van Rossum et al., 2020; Statistics Netherlands, 2024c). The survey reinforces the notion that a disparity exists between what people say they are willing to do and their actual behaviour. While the majority of respondents indicate that they would (certainly or probably) be willing to implement the proposed exercise regimen, only a third of the total sample report already doing so. A similar disparity emerges in the supplements measure, where about half of respondents who are (certainly or probably) open to daily supplement use stated they already use them. Thus, more so than the public’s willingness to engage in exercise and the use of supplements, the intention-behaviour gap constrains the efficacy of these geroprotective measures in advancing public health (Rhodes and de Bruijn, 2013; Faries, 2016).

Conversely, public willingness was found to be lacking for the less common geroprotective strategies. Just above one-fourth of respondents were (certainly or probably) willing to start an intermittent fasting regimen, or take metformin regularly. The least accepted measure, regular rapamycin use, was rejected (certainly or probably not) by three-fourths of respondents, with as little as two percent stating they certainly would be willing to take rapamycin. Thus, a brief and, for many respondents, first introduction to rapamycin leads predominantly to rejection. Scepticism towards geroprotective measures may be explained partly by unfamiliarity and a lack of knowledge, however, we should be careful not to overestimate the explanatory power of this assumption (Većkalov et al., 2023). Medical research into these geroprotective strategies remains inconclusive about the health- and life-span-related benefits, especially across groups in society (Morsli and Bellantuono, 2021; Zhang et al., 2021; Keys et al., 2022; Cienfuegos et al., 2022). Therefore, the scepticism found in this study and previous research could be partly explained by the ambiguous research results and science communication (Partridge et al., 2011; Smol’kin et al., 2018).

Regarding sociodemographic characteristics, only sex seemed to be linked with the willingness to utilise geroprotective measures. Female respondents were more enthusiastic about supplements, whereas male respondents reacted more positively towards the proposed exercise regimen. The socialization process encouraging men and discouraging women from sports participation is well-studied (Chalabaev et al., 2013). Additionally, female sports and exercise research is vastly underdeveloped (Cowley et al., 2021), potentially leading females to devalue the benefits of exercise for health. For supplement use, no such socialization processes are described in the literature even though the gender divide is consistently reaffirmed (van Rossum et al., 2020). It is possible that increased recommendation of supplement use during pregnancy boosts familiarity and by extension supplement use in pregnant and non-pregnant women (Bailey et al., 2013). However, it does not seem likely this effect is solely responsible for the observed gender divide. Our research implies a male-female divide exists in society based on the perceived benefits and risks of these measures. Considering these strategies are well-known and comparatively common, social acceptance plays a role and an element of “doing gender” could be relevant to both. Contradictory to the literature, no association between age and supplement use was found (van Rossum et al., 2020; Bailey et al., 2013), neither was a decreased willingness to participate in exercise amongst older and chronically ill individuals (Loef et al., 2016). Despite sex-based differences in health outcomes for both intermittent fasting and rapamycin (Selvarani et al., 2021; Cienfuegos et al., 2022), sex-based preferences were not associated with these measures. Possibly due to respondents’ being unaware of these discrepancies.

In exploring the role of trust in medical institutions, we found a correlation in the data between the willingness to use metformin and trust. As shown in Figure 1, respondents were predominantly unsure about the use of metformin as a geroprotective agent. Subsequently, this uncertainty could lead people to increasingly rely on their trust in involved actors (i.e., medical institutions) in determining their willingness to use the geroprotective measure (Siegrist, 2000). Of the two drugs included in this study, metformin has more clear scientific backup. It is possible that the combination of a well researched geroprotective drug with trust in medical institutions can lead to more uptake of this drug in the future. Trust in medicine has been shown to be a powerful determinant of adherence and uptake in both preventative and curative healthcare (Hornsey et al., 2020; Pellegrini, 2017). Thus, the geroprotection field of research should be attentive to the importance of trust in emerging geroprotective candidate drugs, such as metformin. Exhibiting trustworthiness could be a valuable focal point for scientists and companies, to generate initial support for unfamiliar geroprotective strategies. Notably, no correlation between trust in medical institutions and rapamycin was found, suggesting the close to uniform rejection of rapamycin indicates less uncertainty and therefore less need to rely on trust to reduce the complexity of the risk management decision (Siegrist, 2000).

The current study holds some limitations and some findings that should be interpreted with caution. First, direct comparison of different measures can become convoluted, due to the multiple shifting components of each measure in regards to required time investment, perceived price, or harmful side-effects. For example, the difference between the willingness to adopt 3 h of endurance exercise and 2 h of weight training weekly into daily life versus one supplement a day might be determined by multiple practical or social confounders, other than a purely health benefits-based evaluation. However, these confounders do not invalidate the analysis, as they are important in determining the willingness to implement geroprotective interventions into daily life. Furthermore, the selection of the measures used in this study is meant to reflect the width of possible geroprotective interventions that have shown potential for disease prevention or improvement. Not all interventions selected possess equal amounts of evidence for their effectiveness (e.g., exercise vs. supplements) and certain interventions may be more present in the public eye (e.g., supplements), which should also be considered. It should be noted that our selection of geroprotective interventions is not exhaustive, and the inclusion of different interventions in subsequent research could aid in the construction of a theoretical framework by which to evaluate how various characteristics of interventions influence the public’s willingness to use geroprotective measures.

Second, as shown in Table 2, age, subjective health, and living with a chronic condition did not significantly correlate with the willingness to engage in any of the proposed geroprotective measures. However, the sample was relatively young and healthy. It is therefore highly likely that we do not capture the full picture with regard to the impact of age and health on willingness to use various geroprotective measures. Additionally due to the relatively high educational background of the study participants we were unable to explore differences based on education, even though literature suggests this may be an important factor here (Partridge et al., 2011). It would be critical in future studies of this kind to evaluate the influence of education on the willingness to adapt geroprotective measures into daily life. Likewise, it would be of importance to understand to what degree responders in favour of rapamycin or metformin use already possessed knowledge on its geroprotective ability and possessed either formal or informal education on this topic. Trust in medical institutions and the acceptance of innovative health measures likely differentiate between countries and cultures, which urges caution in applying these research results outside of the Netherlands (Priest et al., 2003). Moreover, the sample’s limited size prevented the use of more elaborate statistical analyses that could yield more nuanced and accurate results. Therefore, future research should improve on this by drawing a larger sample, as well as a more versatile sample when it comes to sociodemographic characteristics and geographic area.

In conclusion, exploratory research can only provide a first step in understanding the social acceptance of geroprotection measures. More social research, both quantitative and qualitative, is necessary to provide an evidence-based basis with which the geroprotection research field can weigh the social acceptance of different measures to promote vitality and prevent disease. The current study does, however, clearly illustrate that measures that have been more broadly promoted over time through public health policy are also more accepted and used by respondents. Public health campaigns that aim to increase the use of supplements and exercise may consider the sex differences in their uptake. Additionally, while the large majority of our survey responders were not convinced by the geroprotective measures of intermittent fasting, metformin, and rapamycin, there was nonetheless a sizeable portion (∼30%) willing to start intermittent fasting and metformin use. This finding alone can inspire medical practitioners and policymakers to investigate the potential these measures hold for health promotion in ageing societies. No routine or apparent place in the general healthcare system exists for these geroprotective measures specifically, as medicine use and lifestyle interventions are often restricted to specific diseases or health conditions rather than the general mechanisms of ageing. Alongside policy research, future social research may also want to delve deeper into the role of healthcare professionals and facilitating trust relations in promoting the use of innovative geroprotective measures. Thus, both policy evaluation and additional social research are needed if the geroprotection field is to impact public health, promote vitality, and delay the onset of chronic illness in older age.

## Data Availability

The raw data supporting the conclusion of this article will be made available by the corresponding author, upon reasonable request. The fully untraceable data will be made available without undue reservation.
