# Supplementary material for: Attitudes towards geroprotection: measuring willingness, from lifestyle changes to drug use
Source: Front Aging. 2024 Nov 5;5:1440661. doi: 10.3389/fragi.2024.1440661 (PMC11573782; doi:10.3389/fragi.2024.1440661)
Supplement: Supplementary file 1 [file DataSheet1.docx]

Supplementary Material

**Attitudes towards geroprotection: measuring willingness, from lifestyle changes to drug use**

Sam J. Brouwers*, Georges E. Janssens, Tali Spiegel

*** Correspondence:** Sam J. Brouwers: sam.j.brouwers@gmail.com

**S1.** Questionnaire Innovate Fostering of General Health

[Vragenlijst het innovatief bevorderen van de algemene gezondheid]

Translated questionnaire from Dutch to English. Original Dutch text is indicated in square brackets. Only questions and texts relevant to this analysis are shown. The survey was originally conducted in Dutch.

Through this form we would like to ask your permission to participate in the study "The Innovate Fostering of General Health". People today often approach healthy living differently. This study is interested in how people think about health programs aimed at overall health, and explores different ways to not just live longer, but also fitter and more vitally.

[Middels dit formulier willen we u toestemming vragen om mee te doen aan het onderzoek “Het innovatief bevorderen van de algemene gezondheid”. Mensen benaderen gezond leven tegenwoordig vaak verschillend. Dit onderzoek is geïnteresseerd in hoe gedacht wordt over gezondheidsprogramma's gericht op de algemene gezondheid, en gaat in op verschillende manieren om niet alleen langer, maar ook fitter en vitaler te leven.]

**DEMOGRAPHIC INFORMATION**

**What is your gender?**

[Wat is uw gender?]

• Male

• Female

• Other

**What is your age?**

[Wat is uw leeftijd?]

-- Select number (0 to 100 years) --

**Would you rate your own health, in general, as excellent, good, fair, mediocre, or poor?**

[Zou u uw eigen gezondheid, in het algemeen, beoordelen als uitstekend, goed, redelijk, matig, of zwak?]

• Excellent

• Good

• Fair

• Mediocre

• Poor

**Do you have a chronic condition, or other non-curable illness?**

[Heeft u een chronische aandoening, of andere niet te genezen ziekte?]

• Yes

• No

• I won’t say

**GEROPROTECTIVE MEASURES**

The following questions focus on a few types of health programmes. These programmes are not disease- or group-specific, but target everyone, and aim to improve and prolong overall health and life expectancy.

[Bij de volgende vragen staan enkele soorten gezondheidsprogramma's centraal. Deze programma's zijn niet ziekte- of groepsspecifiek, maar zijn gericht op iedereen, en hebben als doelstelling algemene gezondheid en levensverwachting te verbeteren en te verlengen.]

**Exercise & sports**

[Bewegen & sport]

Regular exercise is good for your overall health. For instance, research indicates that sport reduces the risk of cardiovascular disease and osteoporosis, and extends lifespan. In addition, regular exercise lowers the risk of Alzheimer's and diabetes.

Would you be willing to start practising sports and exercising more often (at least 3 hours a week of endurance activity, e.g. running or cycling, and 2 hours a week of weight training)?

[Regulier bewegen is goed voor de algemene gezondheid. Zo duidt onderzoek aan dat sport de kans op hart- en vaatziekten en botontkalking verkleint, en het levensduur verlengt. Daarnaast verlaagt geregeld bewegen de kans op alzheimer en diabetes.

Zou u bereid zijn te beginnen met meer sporten en bewegen (minimaal 3 uur per week duuractiviteit, bijvoorbeeld hardlopen of wielrennen, en 2 uur per week gewichtstraining)?]

• I am already doing this

• Certainly yes

• Probably yes

• Possibly yes or no

• Probably not

• Certainly not

**Eating pattern**

[Eetpatroon]

Scientific studies have shown that intermittent fasting can have benefits in counteracting health risks in, among others, obesity, diabetes, cardiovascular disease, cancer and dementia. Studies with animals show that intermittent fasting improves health throughout the life cycle, but studies on intermittent fasting in humans are often only months long, and therefore cannot demonstrate this.

Would you be willing to start intermittent fasting (eating at least 5 days a week for a maximum of 8 hours a day)? For example, eating nothing outside the period from 11 am to 7 pm from Monday to Friday.

[Wetenschappelijke studies hebben aangetoond dat periodiek vasten voordelen kan hebben bij het tegengaan van gezondheidsrisico’s bij onder meer obesitas, diabetes, hart- en vaatziekten, kanker en dementie. Studies met dieren tonen aan dat periodiek vasten de gezondheid verbetert gedurende de hele levensloop, maar onderzoeken naar periodiek vasten bij mensen zijn vaak slechts maanden lang, en kunnen dit daarom niet aantonen.

Zou u bereid zijn te beginnen met periodiek vasten (minstens 5 dagen per week maximaal 8 uur per dag eten)? Bijvoorbeeld maandag tot vrijdag niks eten buiten de periode van 11 uur 's ochtends tot 7 uur 's avonds.]

• I am already doing this

• Certainly yes

• Probably yes

• Possibly yes or no

• Probably not

• Certainly not

**Supplements**

[Supplementen]

Scientific insight shows that taking supplements can have a positive impact on health. Below is an example of a supplement.

Omega 3 fatty acids are common in oily fish, and some vegetable oils. Studies point out that fatty acids reduce the risk of heart failure and heart attacks. In addition, they have anti-inflammatory effects, which help to recover from illnesses and injuries. You can also buy omega-3 fatty acids supplements made in a completely vegan way.

Would you be willing to start taking an omega 3 fatty acids capsule daily, or any other supplement with proven health benefits? Think magnesium or vitamin D.

[Uit wetenschappelijke inzichten blijkt dat het innemen van supplementen een positieve invloed kan hebben op de gezondheid. Hieronder staat een voorbeeld van een supplement.

Omega 3-vetzuren komen veel voor in vette vis, en sommige plantaardige oliën. Studies wijzen erop dat de vetzuren het risico op hartfalen en hartaanvallen verkleinen. Daarnaast werken ze ontstekingsremmend, wat helpt bij het herstellen van ziekten en verwondingen. Ook zijn er omega 3-vetzuren supplementen te koop die op een volledig veganistische manier zijn gemaakt.

Zou u bereid zijn te beginnen met het dagelijks innemen van een omega 3-vetzuren capsule, of een ander supplement met aantoonbare gezondheidsvoordelen? Denk hierbij aan magnesium of vitamine D.]

• I am already doing this

• Certainly yes

• Probably yes

• Possibly yes or no

• Probably not

• Certainly not

Besides eating patterns, exercise and supplements, there is another research field of general health, which is making progress in the field of pharmaceuticals and medication. Two types of medication are described below as examples. Both are intended to improve overall health, and slow down the ageing process. In addition, these medications are not just for the sick, but rather for everyone of adult age. Older, middle-aged, and young adults.

[Naast eetpatroon, beweging en supplement is er nog een onderzoeksveld van de algemene gezondheid, waarbij stappen worden gezet op het gebied van farmaceutica en medicatie. Hieronder staan twee soorten medicatie beschreven als voorbeelden. Beiden zijn bedoeld om de algemene gezondheid te verbeteren, en het ouderdomsproces af te remmen. Daarnaast is deze medicatie niet alleen bestemd voor zieken, maar juist voor iedereen van volwassen leeftijd. Ouder, op middelbare leeftijd, en jongvolwassen.]

**Metformin**

[Metformine]

Metformin is the most widely prescribed drug against type 2 diabetes worldwide. However, research in animals also suggests that metformin reduces the risk of cancer, and prevents dementia, even in individuals without diabetes. Animals on metformin medication lived up to more than 10% longer in some studies. However, metformin does sometimes have side effects. For instance, it can lead to vitamin B12 deficiency, and there are often mild intestinal symptoms that gradually disappear.

Despite the risk of side effects, would you be willing to start taking metformin (one pill a week) if it gives health- and life expectancy-related benefits?

[Metformine is het wereldwijd meest voorgeschreven medicijn tegen diabetes type 2. Onderzoek in dieren duidt er echter ook op dat metformine het risico op kanker verkleint, en dementie tegengaat, ook in personen zonder diabetes. Dieren met metformine medicatie leefden in sommige studies tot meer dan 10% langer. Wel heeft metformine soms bijwerkingen. Zo kan het leiden tot een vitamine B12 tekort, en zijn er vaak milde darmklachten die geleidelijk verdwijnen.

Zou u, ondanks de kans op bijwerkingen, bereid zijn te beginnen met het innemen van metformine (één pil per week) als dit gezondheids- en levensverwachting gerelateerde voordelen geeft?]

• Certainly yes

• Probably yes

• Possibly yes or no

• Probably not

• Certainly not

**Rapamycin**

[Rapamycine]

Rapamycin is a drug used to suppress the immune system in organ transplants, but also a potential drug for improving general health. Scientific studies on rapamycin have largely been conducted in mice. Research indicates that rapamycin delays or prevents dementia, hearing loss, heart muscle disease, osteoporosis, and more age-related diseases. For example, administering rapamycin led to a 26% lifespan extension in mice. This is more than with metformin. However, rapamycin does have side effects. It is often associated with oral mucosal inflammation, gastrointestinal complaints, and a weakened immune system. In addition, rapamycin also leads in some cases to too much cholesterol in the blood, rashes and eczema.

Despite the risk of side effects, would you be willing to start taking rapamycin (one pill a week) if it gives health- and life-expectancy-related benefits?

[Rapamycine is een medicijn wat gebruikt wordt om het immuunsysteem te onderdrukken bij orgaan transplantaties, maar ook een potentieel medicijn voor het verbeteren van de algemene gezondheid. Wetenschappelijke studies naar rapamycine zijn grotendeels bij muizen uitgevoerd. Onderzoek duidt aan dat rapamycine dementie, gehoorverlies, hartspierziekte, botontkalking, en meer ouderdomsziektes vertraagd of tegengaat. Zo leidde het toedienen van rapamycine tot een 26% levensduur verlenging bij muizen. Dit is meer dan bij metformine. Wel heeft rapamycine bijwerkingen. Het gaat vaak gepaard met mondslijmvlies ontsteking, maag en darm klachten, en verzwakte afweer. Daarnaast leidt rapamycine in sommige gevallen ook tot te veel cholesterol in het bloed, uitslag en eczeem.

Zou u, ondanks de kans op bijwerkingen, bereid zijn te beginnen met het innemen van rapamycine (één pil per week) als dit gezondheids- en levensverwachting gerelateerde voordelen geeft?]

• Certainly yes

• Probably yes

• Possibly yes or no

• Probably not

• Certainly not

**TRUST IN MEDICAL INSTITUTIONS**

To conclude, there is a question about your trust.

[Afsluitend is er een vraag naar uw vertrouwen.]

**How much trust do you have in the following institutions?**

[Hoeveel vertrouwen heeft u in de volgende instanties?]

Scientists and researchers at universities

[Wetenschappers en onderzoekers aan universiteiten]

• Very much trust

• Much trust

• Some trust

• Little trust

• No trust

Pharmaceutical companies

[Farmaceutische bedrijven]

• Very much trust

• Much trust

• Some trust

• Little trust

• No trust

Doctors and hospital personnel

[Dokters en ziekenhuispersoneel]

• Very much trust

• Much trust

• Some trust

• Little trust

• No trust

Your general practitioner and general practice

[Uw huisarts en de huisartsenpraktijk]

• Very much trust

• Much trust

• Some trust

• Little trust

• No trust
